# Multiple calcium sources are required for intracellular calcium mobilization during gastric organoid epithelial repair

**DOI:** 10.14814/phy2.14384

**Published:** 2020-03-08

**Authors:** Kristen A. Engevik, Rebekah A. Karns, Yusuke Oshima, Marshall H. Montrose

**Affiliations:** ^1^ Department of Pharmacology and Systems Physiology University of Cincinnati Cincinnati OH USA; ^2^ Division of Biomedical Informatics Cincinnati Children's Hospital Medical Center Cincinnati OH USA; ^3^ Biomedical Optics Lab Graduate School of Biomedical Engineering Tohoku University Miyagi Japan

**Keywords:** calcium signaling, epithelium, organoid, repair, stomach

## Abstract

Calcium (Ca^2+^) is a known accelerator for gastric wound repair. We have demonstrated in vivo and in vitro that intracellular Ca^2+^ increases in the gastric epithelial cells directly adjacent to a damaged cell, and that this Ca^2+^ rise is essential for the cellular migration that rapidly repairs the epithelium (restitution). While intracellular Ca^2+^ has been shown to be an important signaling factor during epithelial restitution, the source from which this intracellular Ca^2+^ originates remains unclear. Using gastric organoids derived from mice transgenic for a genetically encoded Ca^2+^ indicator, we sought to investigate the potential sources of intracellular Ca^2+^ mobilization. During confocal imaging, photodamage (PD) was induced to 1–2 gastric organoid epithelial cells and epithelial restitution measured simultaneously with changes in intracellular Ca^2+^ (measured as FRET/CFP ratio in migrating cells adjacent to the damaged area). Inhibition of voltage‐gated Ca^2+^ channels (verapamil, 10 µM) or store‐operated calcium entry (YM58483, 20 µM) resulted in delayed repair and dampened intracellular Ca^2+^ response. Furthermore, inhibition of phospholipase C (U73122, 10 µM) or inositol trisphosphate receptor (2‐APB, 50 µM) likewise resulted in delayed repair and dampened Ca^2+^ response. Results suggest both extracellular and intracellular Ca^2+^ sources are essential for supplying the Ca^2+^ mobilization that stimulates repair.

## Graphical abstract

Intracellular calcium mobilization is known to be essential for proper gastric epithelial repair, however the source from which calcium mobilization originates remains unclear. Our work examines intracellular calcium signaling during epithelial repair in response to photodamage of individual cells. Our work demonstrates that gastric organoids exhibit essential parallels with native tissue and provides insight into the complexity of extracellular and intracellular calcium sources needed for intracellular calcium mobilization to occur in specific migrating (restituting) cells juxtaposed to the damaged cell.

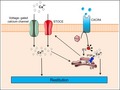

## 1 INTRODUCTION

The epithelial lining of the stomach is part of a protective barrier that is essential to maintain tissue integrity. Despite its robust and multicomponent nature, the gastric mucosal barrier can be breached. Exposure to chemicals or medications, physical insults, local infections by pathogens, and a variety of systemic diseases can lead to disruption of the barrier and damage to the epithelium (Kusters, Vliet, & Kuipers, 2006; Tarnawski, 2005). The epithelium has the ability to quickly repair after injury through a process known as epithelial restitution, which is the initiating step in repair and involves migrating epithelial cells extending lamellipodia over the damaged mucosa to quickly (<1 hr) cover small erosions and reestablish an intact epithelium. Restitution allows for the rapid replacement of damaged cells without compromising the epithelial barrier, thereby preventing further damage to the mucosa. Damage to the gastric mucosa can be mild and readily repaired, or extensive and potentially result in ulcers. As a result, it is important to understand the mechanisms that mediate gastric restitution. While several factors have been identified to be essential for proper restitution, including calcium (Ca^2+^) mobilization (Aihara et al., 2013), actin polymerization (Aihara, Matthis, et al., 2018), and trefoil factors (Engevik et al., 2019; Xue, Aihara, Podolsky, Wang, & Montrose, 2010; Xue, Aihara , Wang, & Montrose, 2011), much of the signaling mechanism driving these events remain unclear. The development of strategies to reduce ulcer incidence or accelerate the healing process represents an important goal for gastric research.

Calcium (Ca^2+^) has been known as an effector of gastric wound repair since 1985 (Cheng et al., 2001; Critchlow et al., 1985; Takeuchi, Nobuhara, & Okabe, 1985). It has been observed in cultured rabbit gastric epithelial cells that intracellular Ca^2+^ is present in significantly higher amounts in migrating cells at the edge of a scratch wound 2 hr following damage (Ranta‐Knuuttila et al., 2002). Furthermore, in these cells, treatment with verapamil (a calcium channel blocker), calphostin‐C (PKC inhibitor), and calmidazolium (calcium/calmodulin complex inhibitor) significantly inhibited cell migration speed observed at 24 hr following monolayer wounding (Ranta‐Knuuttila et al., 2002). In vivo in mice, both intracellular and extracellular Ca^2+^ have been shown to be essential for proper gastric wound repair (Aihara et al., 2013). However, the mechanistic basis of Ca^2+^ mobilization in healthy tissue has largely been unexplored due to past limitations of Ca^2+^ sensors and difficulty in monitoring intracellular Ca^2+^ or wound repair in real time.

Recent advancements in high‐resolution microscopy and genetically encoded Ca^2+^ indicators present the opportunity to monitor Ca^2+^ mobilization during epithelial repair. Using live imaging of gastric organoids derived from mice with a genetically encoded Ca^2+^ sensor, Yellow Cameleon Nano15 (YC Nano) transgenic mouse (Engevik et al., 2019; Horikawa et al., 2010; Oshima et al., 2014), we sought to investigate the signaling cascade behind Ca^2+^ mobilization during gastric epithelial repair. The gastric organoid culture system contains the cell types found in normal native tissue (Bartfeld et al., 2015; Schumacher et al., 2015). We have previously shown using YC Nano‐derived gastric organoids that Ca^2+^ is mobilized at the leading edge of a photodamage‐induced wound (Engevik et al., 2019). We also observed that organoids retain properties of repair found in native mouse gastric epithelium, including a dependence on Ca^2+^ mobilization, trefoil factor 2 and sodium exchanger 2 for efficient restitution (Engevik et al., 2019). Using gastric organoids derived from YC Nano transgenic mice, we now seek to investigate the potential sources of intracellular Ca^2+^ mobilization during repair.

## 2 RESULTS

### 2.1 Identifying potential calcium targets in gastric organoids

**Figure 1:**
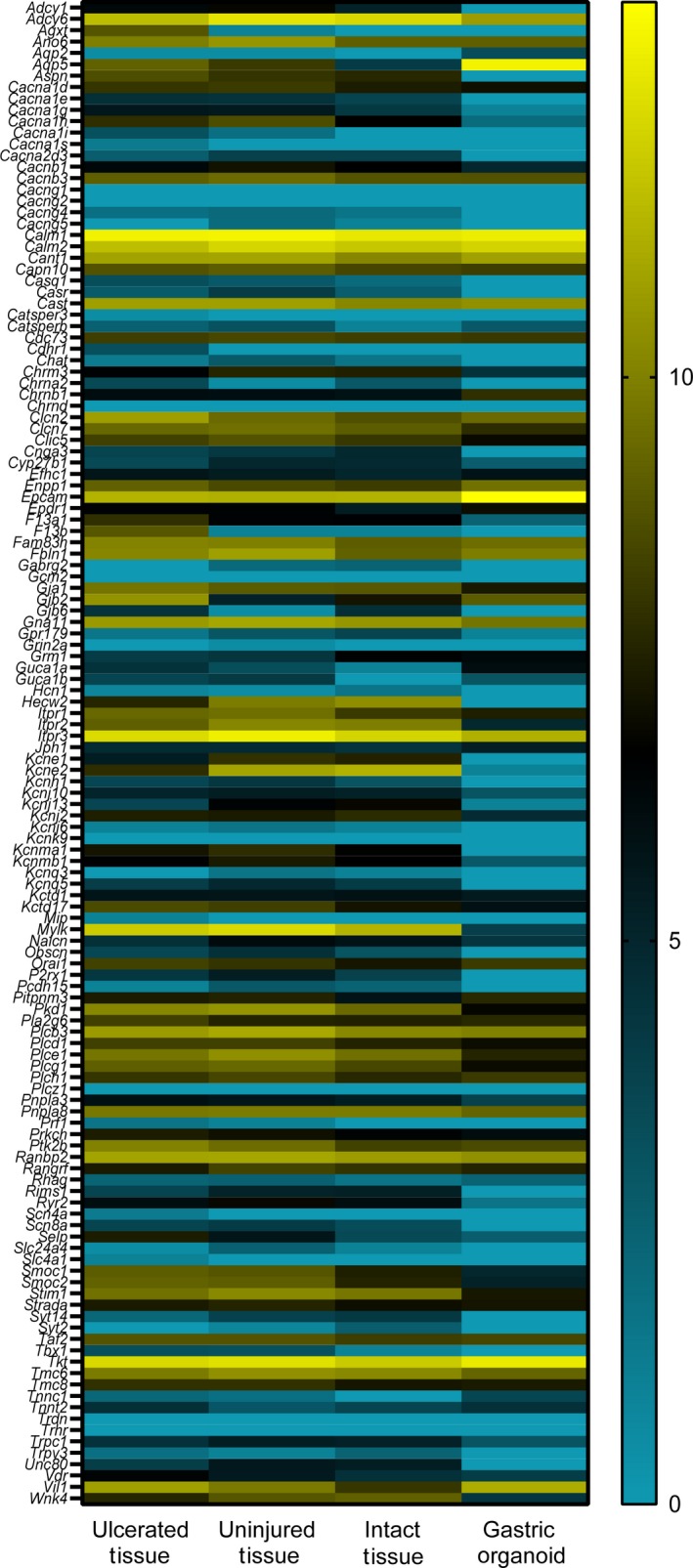
Heatmap of hierarchical clustering for 125 potential targets involved in calcium signaling within gastric organoids and native tissue. Histogram in color key indicates the distribution of RNA expression values; low expression is shown in black, and higher expression is shown in yellow. Heatmap is based upon RNA sequence data set acquired from Engevik et al., *CMGH*, 2016 http://www.ncbi.nlm.nih.gov/geo/query/acc.cgi?acc=GSE73336

To identify potential Ca^2+^‐signaling‐related genes present in both mouse corpus tissue and corpus‐derived gastric organoids, we utilized previously published RNA sequence data from mice (Engevik et al., 2016). We compared intact mouse corpus (never exposed to injury), regions of corpus ulcerated via acetic acid injury, adjacent uninjured regions of corpus, and mouse corpus‐derived gastric organoids. Data are presented as log_2_ of the raw Transcripts per Million (TPM) (Figure 1; Table 1). We identified 132 gene targets whose annotations indicated they were involved in Ca^2+^ signaling (Figure 1; Table 1). Among these 132 gene targets, when the top 50 highest expressed genes are rank ordered in either organoids or tissues, there was notable concordance of 39 shared gene targets (Table 2). Among the 39 shared genes were calmodulin 1 and 2 (calm1, calm2), inositol trisphosphate receptor (IP_3_R) type 1 and 3 (itpr1, itpr3), calcium voltage‐gated channel subunit beta 3 (cacnb3) and subunit alpha 1 D (cacna1d), stromal interaction molecule 1 (stim1) and phospholipase C (PLC) family members beta 3 (plcb3), delta 1 (plcd1), epsilon 1 (plce1), and eta 1 (plch1). Interestingly, organoids exhibited a similar pattern of gene expression of these calcium‐related transcripts to uninjured, intact and ulcerated tissues (Figure 1; Table 1). Of the Top 50 expressed genes for each group, 39 genes (78%) were common between organoids and all tissue types (uninjured, intact, and ulcerated tissue), with only 7 genes (14%) found to be present in tissue, but absent in organoids (Table 2). It is possible that these genes are present in nonepithelial cell types and thus not present in organoids. Additionally, we observed that one gene (2%), gjb2, was present in the ulcerated tissue and in organoids, but not present in the top 50 expressed genes of uninjured and intact tissue; although it was expressed by both uninjured and intact tissue. We also found that 2 genes (4%; hecw2 and kcne1) were expressed in the top 50 genes of uninjured and intact tissue, but not present in the top 50 genes in the organoids and ulcerated tissue; although both were expressed by organoids and ulcerated tissue. These data indicate that the profile of calcium‐related genes are similar between organoids and tissue. However, it should also be noted that there were Ca^2+^ targets that were highly expressed in one sample but at low expression in the other, demonstrating that the organoids are not a perfect representation of native tissue. Among the highest expressions found in both organoid and tissue sample, we chose to evaluate the functional role of voltage‐gated Ca^2+^ channels, PLC, and IP_3_R, as well as the store‐operated Ca^2+^ entry (SOCE) pathway based upon expression of SOCE‐associated genes (stim1, orai1).

**Table 1:** Fifty most highly expressed calcium targets shown in order of expression for each sample type

#	Ulcerated	Uninjured	Intact	Organoid
1	Calm1	Calm1	Calm1	Epcam
2	Itpr3	Itpr3	Adcy6	Aqp5
3	Tkt	Adcy6	Itpr3	Calm1
4	Mylk	Tkt	Tkt	Tkt
5	Adcy6	Mylk	Calm2	Calm2
6	Calm2	Calm2	Mylk	Itpr3
7	Epcam	Epcam	Epcam	Vil1
8	Ranbp2	Plcb3	Kcne2	Cant1
9	Cant1	Gna11	Ranbp2	Adcy6
10	Vil1	Ranbp2	Gna11	Cast
11	Cast	Cant1	Hecw2	Ranbp2
12	Plcb3	Kcne2	Plcb3	Plcb3
13	Gna11	Cast	Tmc6	Fbln1
14	Clcn2	Fbln1	Cast	Gna11
15	Pkd1	Ano6	Cant1	Enpp1
16	Fbln1	Tmc6	Itpr2	Fam83h
17	Gjb2	Pkd1	Pnpla8	Clcn2
18	Tmc6	Plce1	Stim1	Pnpla8
19	Ano6	Itpr2	Plce1	Tmc6
20	Fam83h	Vil1	Pkd1	Ano6
21	Gja1	Stim1	Fbln1	Gjb2
22	Pnpla8	Hecw2	Wnk4	Cacnb3
23	Ptk2b	Pnpla8	Ano6	Ptk2b
24	Plce1	Fam83h	Fam83h	Taf2
25	Stim1	Clcn2	Clcn7	Capn10
26	Itpr1	Clcn7	Gja1	Orai1
27	Clcn7	Itpr1	Cacnb3	Plch1
28	Aqp5	Ptk2b	Clcn2	Cdc73
29	Plcg1	Cacnb3	Plcg1	Chrnb1
30	Itpr2	Plcg1	Capn10	Clcn7
31	Smoc2	Gja1	Ptk2b	Pitpnm3
32	Enpp1	Smoc2	Cdc73	Pla2g6
33	Cacnb3	Wnk4	Taf2	Plce1
34	Smoc1	Capn10	Enpp1	Rangrf
35	Taf2	Smoc1	Itpr1	Itpr1
36	Capn10	Taf2	Clic5	Tmc8
37	Kctd17	Clic5	Vil1	Gja1
38	Aspn	Cacna1h	Rangrf	Stim1
39	Agxt	Enpp1	Kcnj2	Strada
40	Plcd1	Plch1	Plch1	Cacna1d
41	Orai1	Cdc73	Aspn	Epdr1
42	Plch1	Rangrf	Plcd1	Plcd1
43	Pla2g6	Plcd1	Smoc2	Plcg1
44	Clic5	Aspn	Kcne1	Clic5
45	F13b	Aqp5	Pla2g6	Pkd1
46	Cdc73	Kctd17	Chrm3	Grm1
47	Cacna1d	Cacna1d	Smoc1	Prkch
48	F13a1	Kcne1	Cacna1d	Guca1a
49	Tmc8	Orai1	Tmc8	Kctd17
50	Kcne2	Kcnma1	Orai1	Efhc1

**Table 2:** Fifty most highly expressed calcium targets categorized by the sample types that share the same transcripts in their list (results shown alphabetically)

All	Tissue, but not organoids	Injured only	Injured & organoids	Uninjured & intact	Organoids only
Adcy6	Aspn	F13a1	Gjb2	Hecw2	Chrnb1
Ano6	Itpr2	F13b		Kcne1	Epdr1
Cacna1d	Kcne2				Grm1
Cacnb3	Mylk				Guca1a
Calm1	Smoc1				Pitpnm3
Calm2	Smoc2				Prkch
Cant1	Wnk4				Strada
Capn10					
Cast					
Cdc73					
Clcn2					
Clcn7					
Clic5					
Enpp1					
Epcam					
Fam83h					
Fbln1					
Gja1					
Gna11					
Itpr1					
Itpr3					
Kctd17					
Orai1					
Pkd1					
Pla2g6					
Plcb3					
Plcd1					
Plce1					
Plcg1					
Plch1					
Pnpla8					
Ptk2b					
Ranbp2					
Stim1					
Taf2					
Tkt					
Tmc6					
Tmc8					
Vil1					

### 2.2 Voltage‐gated calcium channels are essential for intracellular calcium mobilization during repair

**Figure 2:**
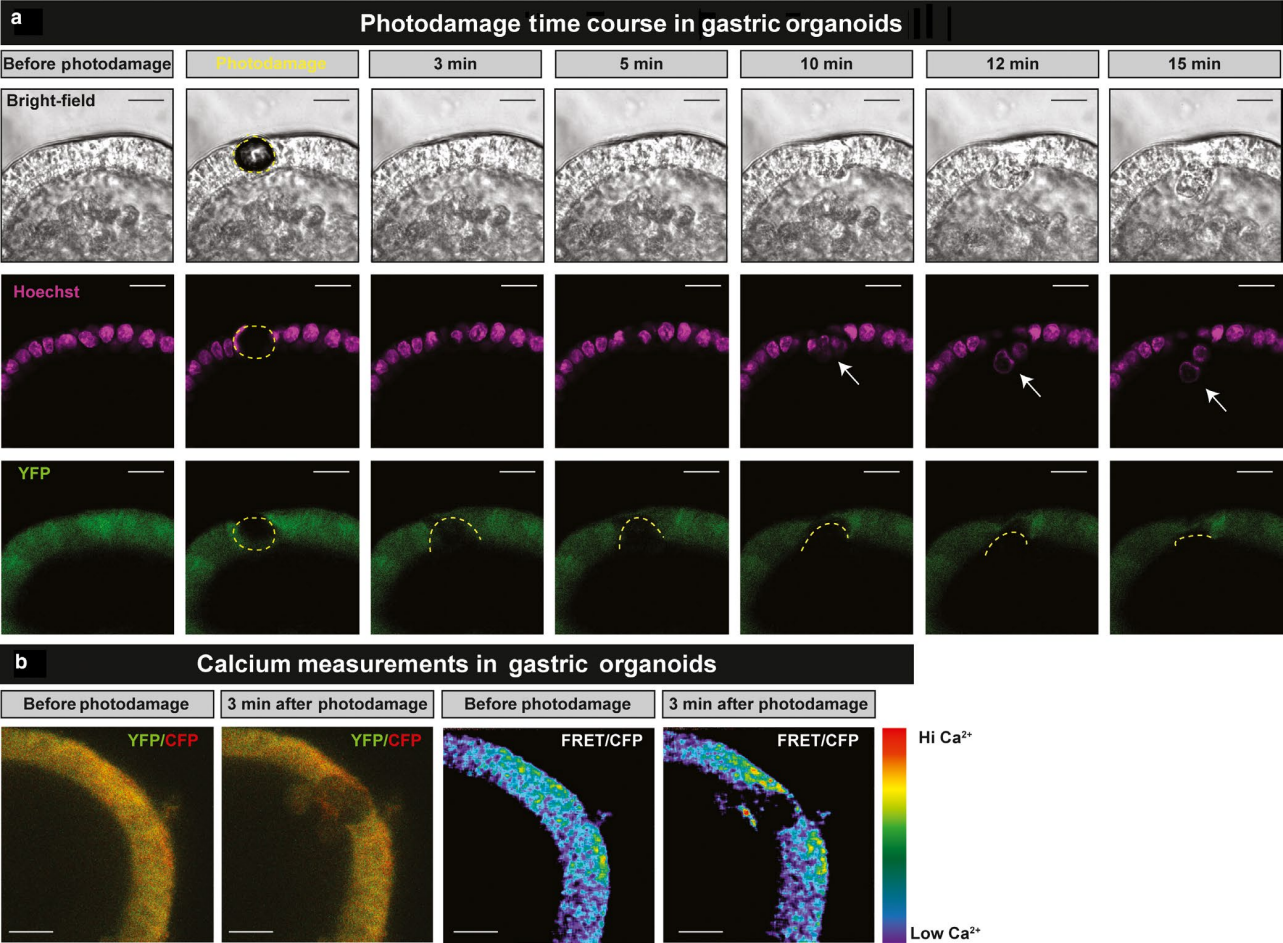
Photodamage and repair in gastric organoids over time. (a) Representative confocal images of YC Nano corpus‐derived gastric organoids before, during and following photodamage. Yellow dashed lines highlight damage zone. (b) Representative FRET/CFP ratio median filter image of YC Nano gastric organoid under 458 nm excitation before and 3 min after photodamage. Scale bar = 10 µm

**Figure 3:**
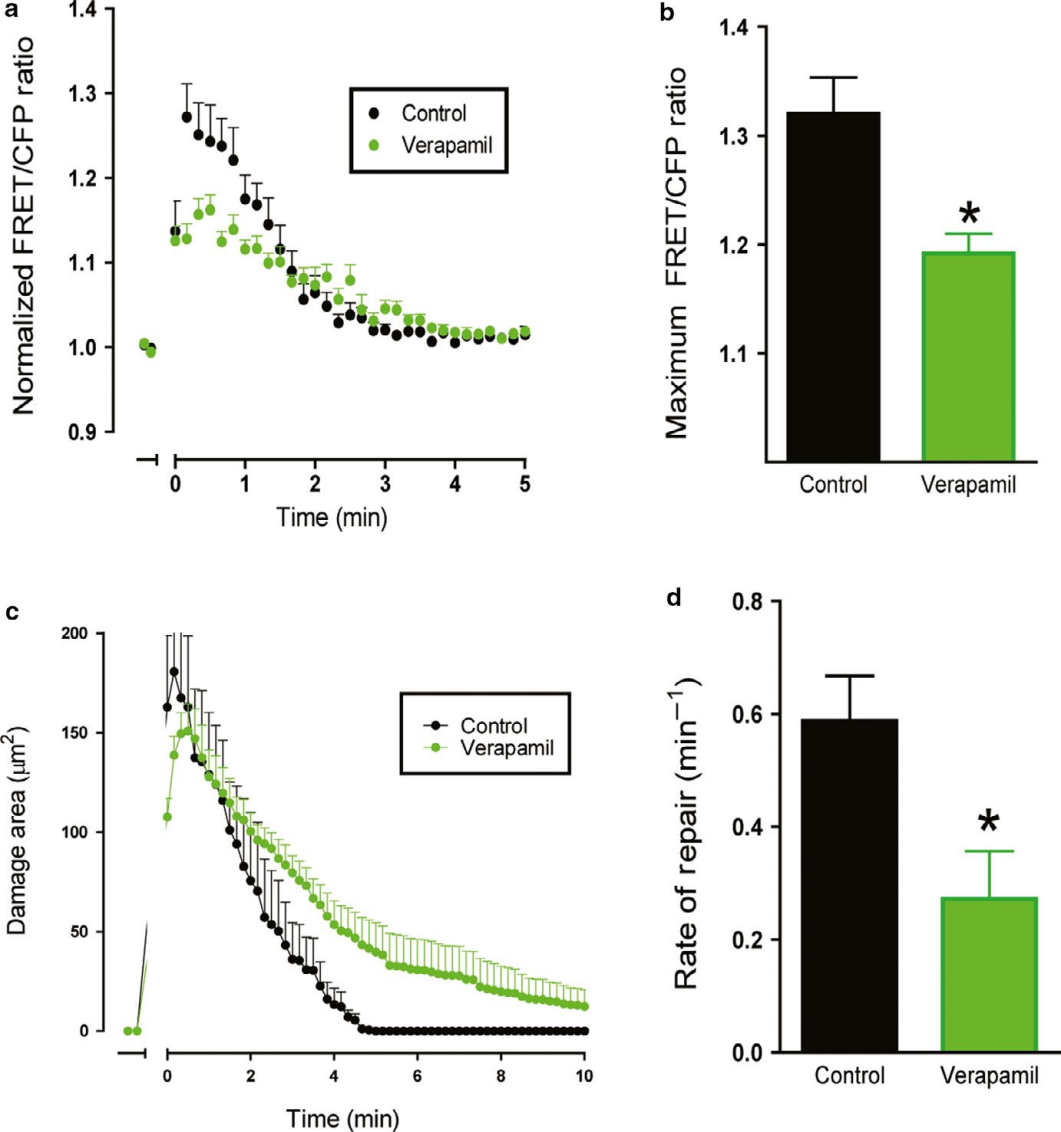
Effect of voltage‐gated calcium channel blocker on calcium mobilization and repair. Fluorescence of YC Nano gastric organoids was imaged over time. Verapamil (10 µM) was added to organoid medium 1 hr prior to experimentation. In time course, PD occurred at t = 0 min. (a) Measurement of normalized FRET/CFP ratio of lateral membrane region of cells adjacent to the damage site comparing control (black) and verapamil supplemented gastric organoids (green). Comparison of the maximum FRET/CFP ratio from panel B between control (black, *n* = 3) and verapamil‐treated (green, *n* = 5) gastric organoids (*p < .05). (c) Damage area measured in control (black, *n* = 3) and verapamil supplemented gastric organoids (green, *n* = 5) over time. (d) Comparison of rate of repair control (black, *n* = 3) and verapamil‐treated (green, *n* = 5) gastric organoids (*p < .05)

To address the role of different calcium stores in gastric epithelial repair, we generated organoids from the corpus of YC Nano Ca^2+^ sensor mice and monitored repair by live imaging (Figure 2). Using 2‐photon microscopy, we are able to localize photodamage to the nucleus of 1–2 cells (stained by Hoechst 33342 and identified by dashed yellow lines), and over a period of 15 min we observed a decrease in the area of damage, with migration of neighboring cells and expulsion of the dead cell into the lumen of the organoid (Figure 2a). Using organoids from the YC Nano mice, we measured intracellular Ca^2+^ changes within intact cells neighboring the damage site via ratiometric imaging (Figure 2b). Verapamil was applied 1 hr prior to photodamage (PD) by a brief exposure to high intensity 840 nm light (two‐photon light absorption). Following PD, verapamil (10 µM) dampened Ca^2+^ mobilization compared to the control (Figure 3a). Significant blunting of the Ca^2+^ signaling was observed within cells adjacent to the damage site, where the control FRET/CFP ratio peak was 1.32 ± 0.03 compared to a FRET/CFP ratio peak of 1.19 ± 0.02 (*n* = 3) in the presence of verapamil (*n* = 5, *p* < .05) (Figure 3b). Furthermore, verapamil significantly delayed repair rate (0.27 ± 0.08 min^−1^, *n* = 5) compared to the control repair rate (0.59 ± 0.08 min^−1^, *n* = 3, *p* < .05) (Figure 3c,d). These data suggest voltage‐gated Ca^2+^ channels contribute to the intracellular Ca^2+^ response during repair, implicating uptake of extracellular Ca^2+^ as one source of mobilized Ca^2+^. Results support prior results in native tissue (Aihara et al., 2013), and thereby confirm the use of gastric organoids as a valid model to elucidate Ca^2+^ signaling pathways during gastric repair.

### 2.3 Store operated calcium entry is essential for calcium mobilization during restitution

**Figure 4:**
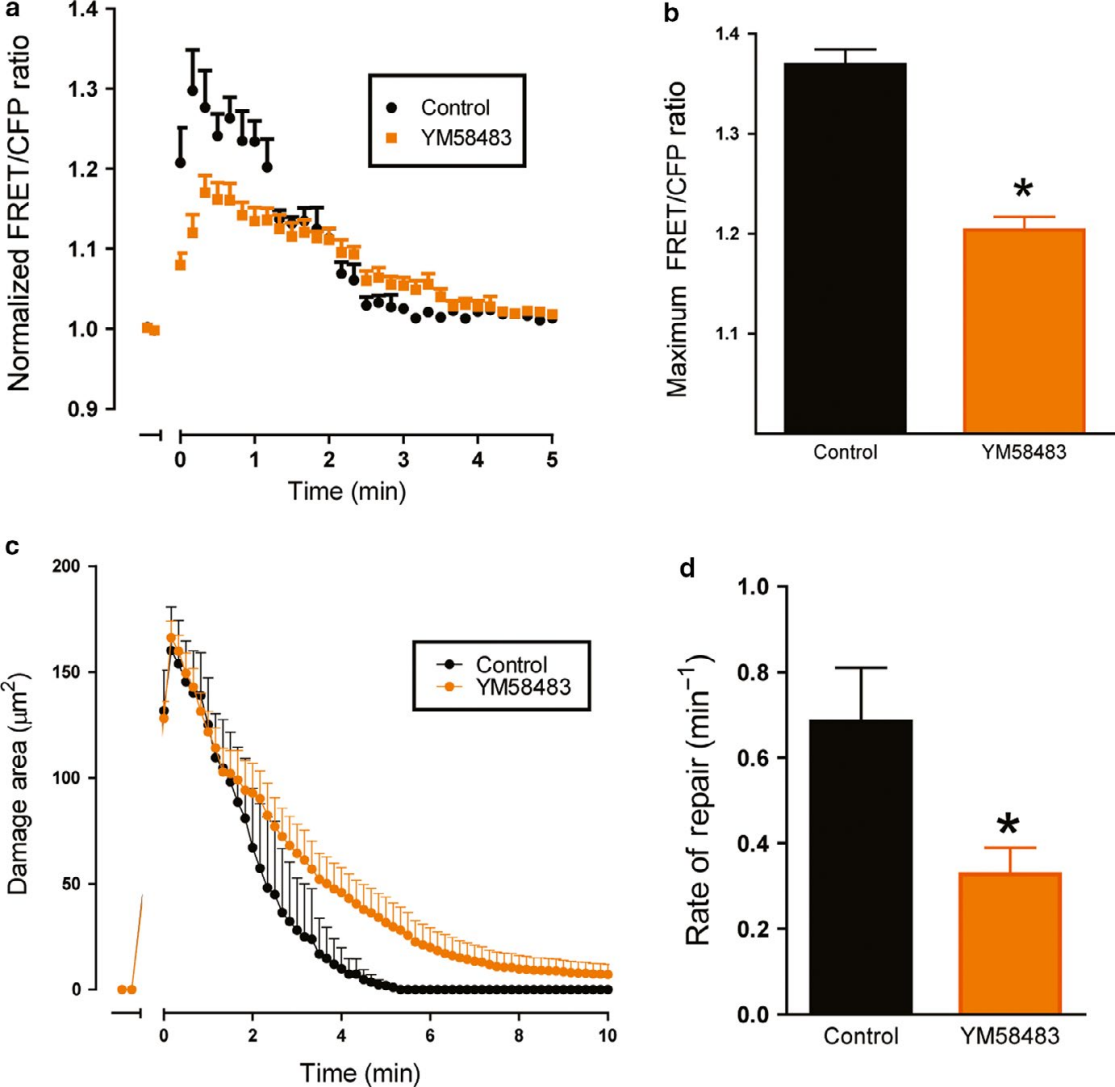
Effect of store‐operated calcium entry inhibition on calcium mobilization and repair. Fluorescence of YC Nanogastric organoids was imaged over time. YM58483 (20 µM) was added to organoid medium 1 hr prior to experimentation. In time course, PD occurred at t = 0 min. (a) Measurement of normalized FRET/CFP ratio of lateral membrane region of cells adjacent to the damage site comparing control (black) and YM58483 supplemented gastric organoids (orange). Comparison of the maximum FRET/CFP ratio from panel B between control (black, *n* = 3) and YM58483‐treated (orange, *n* = 4) gastric organoids (*p < .05). (c) Damage area measured in control (black, *n* = 3) and YM58483 supplemented gastric organoids (orange, *n* = 7) over time. (d) Comparison of rate of repair control (black, *n* = 3) and YM58483‐treated (orange, *n* = 7) gastric organoids (*p < .05)

To further test the role of extracellular Ca^2+^ as an essential source to aid in Ca^2+^ mobilization during gastric repair, an inhibitor of store operated Ca^2+^ entry (SOCE, YM58483, 20 μM) was added to the media 1 hr prior to PD. The presence of YM58483 dampened the Ca^2+^ mobilization following PD (Figure 4a), resulting in a significantly decreased maximum FRET/CFP ratio at 1.2 ± 0.01 (*n* = 4) compared to the control FRET/CFP peak at 1.37 ± 0.02 (*n* = 3, *p* < .05) (Figure 4b). Additionally, inhibition of SOCE significantly delayed the rate of repair, reducing from a control 0.68 ± 0.13 min^−1^ (*n* = 3, *p* < .05) to 0.33 ± 0.06 min^−1^ (*n* = 4) (Figure 4c,d). These data further suggest the role of extracellular Ca^2+^ contributing to the intracellular calcium response during repair.

### 2.4 Phospholipase C (PLC) pathway is necessary for intracellular calcium mobilization during repair

**Figure 5:**
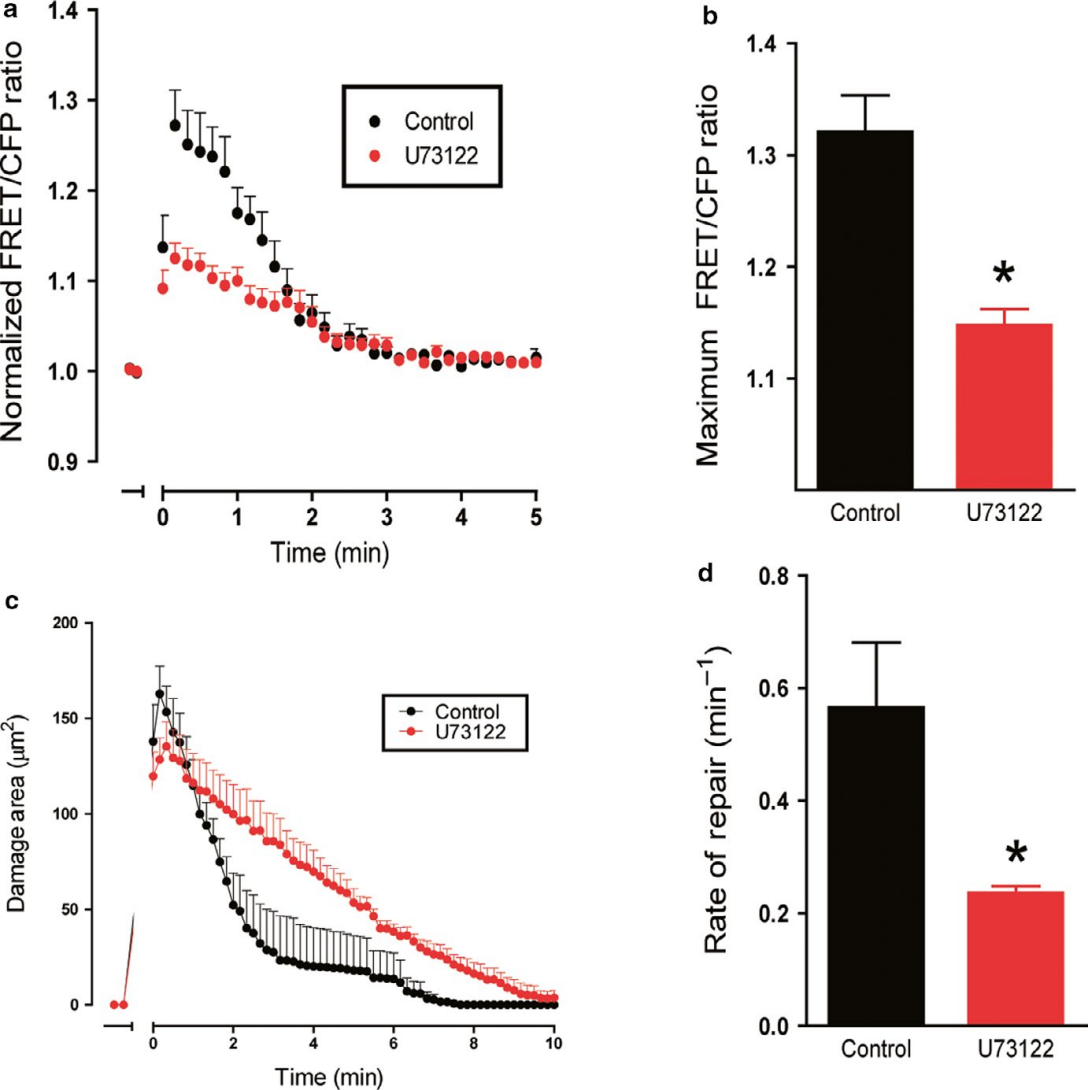
Effect of Phospholipase C inhibition on calcium mobilization and repair. Fluorescence of YC Nanogastric organoids was imaged over time. U73122 (10 µM) was added to organoid medium 1 hr prior to experimentation. In time course, PD occurred at t = 0 min. (a) Measurement of normalized FRET/CFP ratio of lateral membrane region of cells adjacent to the damage site comparing control (black) and U73122 supplemented gastric organoids (red). Comparison of the maximum FRET/CFP ratio from panel B between control (black, *n* = 4) and U73122 (red, *n* = 6) gastric organoids (*p < .05). (c) Damage area measured in control (black, *n* = 4) and U73122 supplemented gastric organoids (red, *n* = 6) over time. (d) Comparison of rate of repair control (black, *n* = 4) and U73122 (red, *n* = 6) gastric organoids (*p < .05)

Next, we sought to examine the role of intracellular Ca^2+^ release in mediating wound repair. We inhibited PLC by adding U73122 (10 μM) into the media and monitored the response following injury. Following PD, U73122 dampened the Ca^2+^ response during repair (Figure 5a), which resulted in a significantly decreased FRET/CFP ratio of 1.15 ± 0.02 (*n* = 6) compared to the control of 1.32 ± 0.03 (*n* = 4, *p* < .05) (Figure 5b). Furthermore, U73122 significantly delayed the rate of repair (0.24 ± 0.01 min^−1^, *n* = 6) compared to control (0.56 ± 0.11 min^−1^, *n* = 4, *p* < .05) (Figure 5c,d). These data confirm previous findings in vivo and in vitro (Aihara et al., 2013; Engevik et al., 2018), identifying PLC as an important player in Ca^2+^ mobilization during epithelial repair.

**Figure 6:**
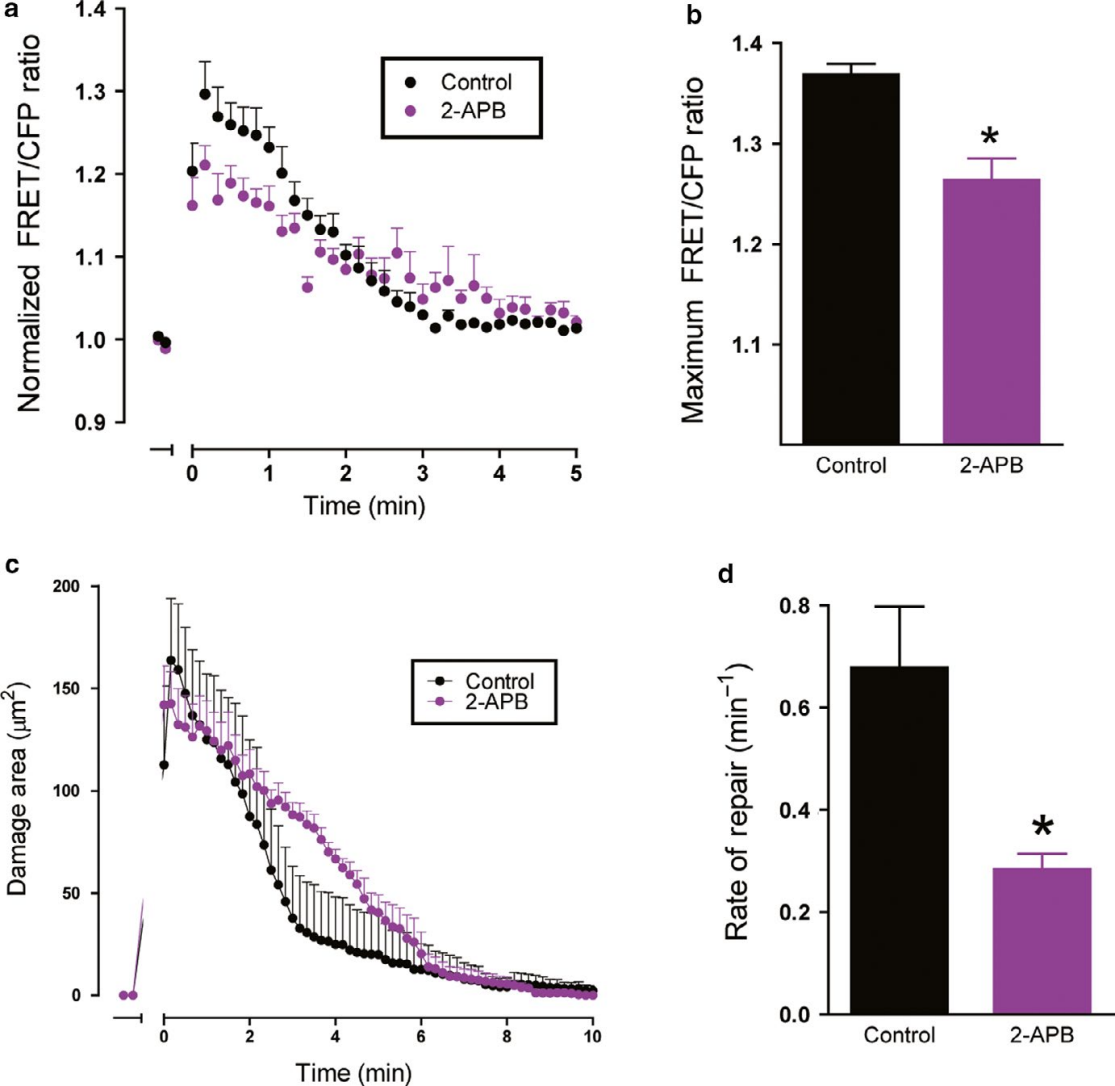
Effect of inositol trisphosphate receptor inhibition on calcium mobilization and repair. Fluorescence of YC Nanogastric organoids was imaged over time. 2‐APB (50 µM) was added to organoid medium 1 hr prior to experimentation. In time course, PD occurred at t = 0 min. (a) Measurement of normalized FRET/CFP ratio of lateral membrane region of cells adjacent to the damage site comparing control (black) and 2‐APB supplemented gastric organoids (purple). Comparison of the maximum FRET/CFP ratio from panel B between control (black, *n* = 3) and 2‐APB (purple, *n* = 4) gastric organoids (*p < .05). (c) Damage area measured in control (black, *n* = 3) and 2‐APB supplemented gastric organoids (purple, *n* = 4) over time. (d) Comparison of rate of repair control (black, *n* = 3) and 2‐APB (purple, *n* = 4) gastric organoids (*p < .05)

PLC is reported to act via a signaling cascade to release of Ca^2+^ from the endoplasmic reticulum (ER) via the downstream receptor inositol triphosphate receptor (IP_3_R) (Putney & Ribeiro, 2000; Sambrook, 1990). According to RNA‐seq data, IP_3_R was highly expressed in both fundic tissue and organoid sample, suggesting IP_3_R as a potential target for Ca^2+^ signaling (Figure 1; Table 1). To test the role of PLC and determine whether the ER release of calcium contributes to repair, we investigated the role of IP_3_R. Gastric organoids were treated with an IP_3_R inhibitor (2‐APB, 50 μM) to test the role of IP_3_R during restitution. Following PD, 2‐APB dampened the Ca^2+^ mobilization (Figure 6a), resulting in a significantly decreased maximum FRET/CFP ratio of 1.26 ± 0.02 (*n* = 4) compared to the control (1.67 ± 0.01, *n* = 3, *p* < .05) (Figure 6b). Furthermore, addition of 2‐APB resulted in a significantly delayed repair rate (0.28 ± 0.03, *n* = 4) compared to the control (0.68 ± 0.12, *n* = 3, *p* < .05) (Figure 6c,d). These data suggest the role of ER‐released Ca^2+^ via IP_3_R as a contributing source to promote epithelial repair.

## 3 DISCUSSION

While signaling in Ca^2+^ dynamics has been well studied in other systems, much remains unclear within the context of gastric epithelial cells, especially during repair. The general role of Ca^2+^ as an effector of gastric wound repair has been established for 30 years (Cheng et al., 2001; Critchlow et al., 1985; Miller & Henagan, 1979; Takeuchi et al., 1985). However, the mechanisms by which Ca^2+^ mobilization affects restitution has yet to be fully elucidated and the source of this Ca^2+^ mobilization remains largely unknown. Herein, we demonstrate that both extracellular and intracellular Ca^2+^ sources contribute to gastric repair in the mouse epithelium, confirming in part of what has been found in vivo (Aihara et al., 2013) as well as demonstrating that gastric organoid experiments can recapitulate in vivo findings. Our work points to the critical role of voltage‐gated and SOCE Ca^2+^ channels (extracellular) and PLC, IP_3_ (intracellular) pathways to promoting repair. We have previously shown in vivo the importance of localized changes in both extracellular and intracellular Ca^2+^ during gastric epithelial repair (Aihara et al., 2013; Aihara & Montrose, 2014) in the first study to demonstrate Ca^2+^ dynamics occurring in native tissue during gastric restitution with a genetically encoded Ca^2+^ indicator. Due to tissue motion in a breathing animal, it is technically challenging to examine Ca^2+^ dynamics in individual cells during the response to single cell damage *(unpublished observations)*. In vivo models are also limited in the ability to apply drugs while avoiding more systemic effects. To circumvent these issues, we used the gastric organoid model. Similar to previous in vivo work, this study found that inhibition of voltage‐gated channels, PLC and IP_3_R, caused significantly decreased Ca^2+^ mobilization in addition to delayed restitution. Our functional findings are parallel to what has been witnessed in vivo, indicating the reliability of gastric organoids to mimic native tissue and confirming the role of both voltage‐gated channels and the PLC pathway during Ca^2+^ driven repair. The concordance of highly expressed calcium‐signaling genes further support these conclusions. This study extends our prior report that restitution in gastric organoids is dependent upon intracellular Ca^2+^ mobilization, acting downstream of several important factors triggering proper repair (Engevik et al., 2019).

Ca^2+^ signaling is a dynamic process involving changes in intracellular Ca^2+^ availability as well as coordination of Ca^2+^ release from surrounding cells following epithelial damage (Sanderson, Charles, Boitano & Dirksen, 1994). As a second messenger, Ca^2+^ can act in several different signaling cascades each of which has a different mechanism by which Ca^2+^ mobilizes. Some of such mechanisms have been shown to cause Ca^2+^ mobilization that is highly localized, brief increases in Ca^2+^ while other pathway mechanisms produce longer‐lasting elevations of Ca^2+^, which often follow oscillations caused by feedback loops within the signaling system (Berridge, 1997). In vivo gastric damage elicits increased gastric luminal Ca^2+^ (Koo, 1994; Takeuchi, Kato, Konaka, & Sugawa, 1999; Takeuchi et al., 1985). While intracellular Ca^2+^ appears to be localized within the cells adjacent to the damaged site, previous studies have been limited in investigating Ca^2+^ mobilization within an in vitro model that closely mirrors in vivo native, noncancerous tissue. Work from Chang‐Graham, Perry, Engevik, Danhof, and, Hyser, 2018 demonstrated, using the GCaMP6s Ca^2+^ sensor in human jejunum enteroids, that rotavirus infection activates dynamic Ca^2+^ signaling through mediation of SOCE and purinergic signaling in infected cells (Chang‐Graham et al., 2018). This speaks of the ability of viruses to utilize innate cellular signaling to induce pathophysiological signaling, while also displaying dynamic Ca^2+^ signaling among cells under normal conditions.

Activation of phospholipase C (PLC) is a known initiator of Ca^2+^‐dependent signaling. Activated PLC cleaves the lipid phosphatidylinositol to release inositol triphosphate (IP_3_) and diacylglycerol (DAG), metabolites that can stimulate Ca^2+^ release from intracellular stores and activate protein kinase C (PKC). PKC is also a conventional target of Ca^2+^‐dependent regulation. Both PLC and PKC have been shown in primary, immortalized, and cancer‐derived cell lines to stimulate epithelial cell migration (Ranta‐Knuuttila et al., 2002; Rao et al., 2007; Saidak et al., 2009). In human gastric cancer (AGS) cells, Ca^2+^ mobilization has been shown to stimulate repair from aspirin and deoxycholate induced damage (Redlak, Power, & Miller, 2007, 2008). A previous study from our lab using mouse GFP‐actin organoids has shown that actin dynamics, as well as coinciding repair of damaged cells, depends upon intracellular Ca^2+^ as well as the activation of PLC (Engevik et al., 2018). The Ca^2+^‐dependent PLC isoforms are known to be localized in gastric surface cells (McGarrity, Peiffer, Neely, Palavarapu, & Koltun, 1996; Miller & Henagan, 1979). While these studies point to the overarching role of endogenous Ca^2+^ in gastric epithelial repair, due to systemic effects and difficulties in vivo, little is known about the signaling needed to regulate and promote Ca^2+^ mobilization.

One known pathway responsible for releasing intracellular Ca^2+^ stores from the endoplasmic reticulum (ER) involves PLC. Either in response to activation via a G‐protein coupled receptor or tyrosine kinase receptor, PLC hydrolyzes the IP_3_ precursor, phosphatidylinositol 4,5‐bisphosphate (PIP_2_) to produce diacylglycerol (DAG) and IP_3_ ( Berridge, 1993). Following hydrolysis of PIP_2_, IP_3_ binds to its receptor IP_3_R which undergoes a conformational change that leads to the mobilization of stored Ca^2+^ from the ER (Mignery & Südhof, 1990). IP_3_R, along with ryanodine receptors (RYRs), are the principal intracellular Ca^2+^ channels responsible for the release of Ca^2+^ from the ER membrane stores (Berridge, 1993). Based upon published RNA sequence data ( Engevik et al., 2016), we tested IP_3_R as an indicator of ER release of Ca^2+^ stores due to its high expression of IP3R type 1 and type 3 in both gastric organoids and tissue. While the RYR family was a potential candidate, the RNA sequence data showed that all RYR family members were among the lowest expression within either gastric organoids or intact tissue. Our data shows that IP_3_R is a necessary component to facilitate intracellular Ca^2+^ mobilization during repair, indicating that the ER plays an important role in supplying Ca^2+^. As SOCE is reported to be activated by release of ER Ca^2+^ stores (Parekh, 2003; Parekh & Penner, 1997), in addition to the high expression of SOCE‐associated genes stim1 in the organoid RNA sequence data, it is possible that SOCE acts following the release of Ca^2+^ via IP_3_R. However further studies are needed to determine the order within the signaling cascade, as well to investigate the effects of repeated damage on restitution.

Overtime, gastric organoids exhibit loss of their cell lineage markers (Schumacher et al., 2015). Work by the Zavros laboratory has demonstrated that long‐term culture of gastric organoids decreases the percentage of parietal, endocrine, chief, and surface pit cells while expanding stem cell markers CD44 and Lgr5 (Schumacher et al., 2015). This work also identified that day 4 gastric organoids more closely mirror tissue than organoids cultured for 7 to 12 days. We have attempted to address this limitation in our work by using freshly generated gastric organoids that have never been passaged, at day 4–5 postgeneration. It has also been shown that co‐culture of gastric organoids with immortalized stomach mesenchymal cells (ISMCs) rescues organoid stem cell expansion (Schumacher et al., 2015). While this co‐culture technique is not conducive to high resolution live cell imaging, in the future it would be worthwhile to compare the repair rates of co‐culture versus monoculture gastric organoids.

Although organoids contain a variety of cell types, we cannot identify them via simple light microscopy and have randomly selected cells for damage. Using gastric organoids from a variety of mouse lines (C57B6J, human GFP‐actin (HuGE), and YC‐Nano), we have observed that all cells respond to damage in a similar manner (Aihara, Medina‐Candelaria, et al., 2018; Engevik et al., 2019). Regardless of the cell type damaged, we consistently observe migration of neighboring cells that cover the denuded area and expulsion of the dead cell in a series of event which conclude by 15–20 min. This rapid and similar regeneration profile suggests that the restitution process may be ubiquitous across the epithelium and not necessarily cell specific. This is in line with physiologic needs in vivo, where damage likely occurs to a variety of cell types. However, while our current experiments indicate that all cells mobilize calcium to mediate repair, we cannot assess any heterogeneity in that response related to cell type. Similarly, it will require using human gastric organoids to shed light on whether this phenomenon is a conserved feature that directly relates to the human condition.

A strength of our work is the ability to use inhibitors to assess pathways involved in restitution. In vivo, off target effects of inhibitors and challenges with localized inhibitor concentrations make mechanistic studies more difficult. Gastric organoids allow us to examine these pathways in more depth. The inhibitor doses we used in this study were based upon published in vitro studies, as well as our own prior experience with some drugs in gastric organoids. Since the inhibitors were added 1 hr prior to experiments and the organoids were only exposed at a maximum of 3‐hr exposure to the inhibitors, we did not observe any adverse effects on the organoids in terms of size, cell shedding (apoptosis), or overall organoid structure. Growth rates and histology were not considered due to the relatively short‐term exposure to the drugs. Since calcium is an important physiological response for homeostasis, we anticipate that long‐term exposure to these pharmacologic agents would affect organoid growth, size, and histology. In the future, studies of long‐term inhibitor exposure may be beneficial as patients are often treated with drugs for extended periods.

**Figure 7:**
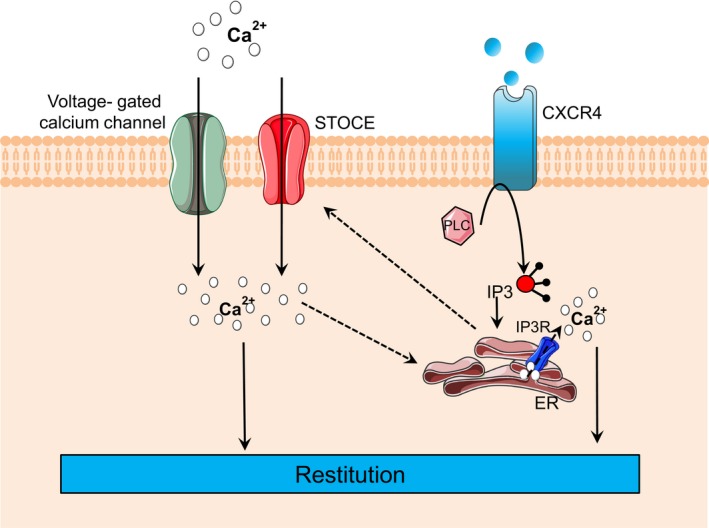
Schematic diagram of calcium mobilization during epithelial restitution. Dotted lines indicate more speculative pathways. *Ca^2+^*, calcium; *SOCE*, store operated calcium entry; *PLC*, phospholipase C; *IP_3_*, inositol trisphosphate; *IP_3_R*, inositol trisphosphate receptor

This study expands our current understanding of Ca^2+^ dynamics by demonstrating the importance of two classes of plasma membrane channels and the role of intracellular ER release in the observed intracellular Ca^2+^ mobilization (Figure 7). These results offer evidence to build future studies that can delve further into the signaling cascade behind gastric epithelial restitution, as well as elucidate the relative contributions and roles of the calcium stores we have identified, with the goal of identifying druggable targets to improve gastric repair in compromised situations.

## 4 MATERIALS AND METHODS

### 4.1 Animal husbandry

Experiments used transgenic mice (C57BL/6 background) expressing the Yellow Cameleon‐Nano15 (YC Nano) Ca^2+^ sensor fluorescent proteins (Engevik et al., 2019; Oshima et al., 2014). Pups were genotyped by genomic PCR as previously described (Bell et al., 1999; Farrell et al., 2002; Schultheis et al., 1998) and used for experimentation at 2–4 months of age. Animals were given standard rodent chow diet and water, both ad libitum. All animal procedures were approved by the Institutional Animal Care and Use Committee of the University of Cincinnati.

### 4.2 Mouse‐derived corpus organoid culture

Gastric organoids were generated from mouse gastric corpus as previously described (Engevik et al., 2018, 2019; Mahe et al., 2013; Schumacher et al., 2015). Isolated gastric epithelium from the corpus was cultured in Matrigel diluted 1:1 in Dulbecco's Phosphate‐Buffered Saline (DPBS) without Ca^2+^ and Mg^2+^ in 8‐well or 2‐well Lab‐Tek chamber with cover glass (Thermo Fisher Scientific) to grow gastric organoids. Gastric organoids were cultured in a 5% CO_2_ incubator at 37°C for 3–4 days prior to experiments.

### 4.3 Induction of two‐photon laser‐induced photodamage

Experiments were performed in organoid culture medium under 5% CO_2_/37°C conditions in a microscope incubation chamber (PeCon, Erbach, Germany) on an inverted confocal microscope (Zeiss LSM 510 NLO) and imaged with a C‐Achroplan NIR 40x objective lens. To assess repair of damage area, images of YFP (excitation 514 nm, emission 535–590 nm) in the gastric organoid were collected simultaneously with transmitted light and a confocal reflectance image (reflecting 730 nm light to show cell/tissue structure). For assessing intracellular Ca^2+^ changes in YC Nanogastric organoids, images of YFP‐FRET (Ti‐Sa laser excitation 840 nm, emission 535–590 nm) and CFP (Ti‐Sa excitation 840 nm, emission 500–530 nm) were collected simultaneously with a transmitted light image. Wavelength selections for Ca^2+^ imaging were guided by previous work with YC sensors (Horikawa et al., 2010; Oshima et al., 2014). In all photodamage (PD) experiments, after collecting a set of control images, a small rectangle region (≈5 µm^2^) of a single cell was repetitively scanned at high Ti‐Sa laser power (730 or 840 nm: 630 mW average) for 500 iterations (requiring ≈3 s).

Experiments examined gastric organoids embedded in Matrigel, located approximately 100–300 μm from the cover glass. Inhibitors were preincubated at least 1 hr prior to experimentation to assure equilibration in Matrigel and inhibitors were kept in the medium during experiments. Inhibitory reagents included: Verapamil (10 μM, Sigma), YM58483 (20 μM, Tocris), U73122 (10 μM, Cayman Chemical), and 2‐APB (50 μM, Tocris). Final DMSO concentration in experiments was <0.1%. Solvent control groups contained 0.1% DMSO added to medium. Vehicle control groups contained either 0.1% DMSO, ddH_2_O, or dPBS added to the medium; vehicle was dependent on the solvent the inhibitors were dissolved in. During experiments with inhibitors, all observed gastric organoids maintained their shape, size and overall integrity, and no increase in shedding (apoptotic) cells was observed compared to untreated gastric organoids. Concentrations were determined based upon in vitro studies and tested to assess effectiveness and potential toxicity.

Damage–repair cycle was measured independently once per gastric organoid, and outcomes from at least three different gastric organoids (derived from at least three animals), were compiled for each experimental protocol.

### 4.4 Image analysis

Damaged area (units of µm^2^) was quantified from the time course of images as described (Aihara et al., 2013, 2014; Engevik et al., 2019; Xue et al., 2010, 2011) using Image J and/or Metamorph software (ver. 6.3, Molecular Devices). The damaged area was measured as the region of cellular loss of YFP fluorescence in YC Nanogastric organoids. In each experiment of YC Nano gastric organoids, we determined the time point displaying maximal damage area and estimated rates of epithelial restitution starting from this time with a single exponential curve fit to the size of damage area over time (Engevik et al., 2018, 2019; Xue et al., 2010). Best fit values of the rate constant were used as estimates of the *rate of repair* (units of min^−1^). Changes in intracellular Ca^2+^ were measured as FRET/CFP ratio using YC Nano gastric organoids. Background images were subtracted from FRET‐YFP and CFP images, the resultant images were divided on a pixel‐by‐pixel basis to calculate the FRET/CFP ratio image. All time course ratio images were then normalized to the averaged predamage baseline images. Regions of interest were determined by transmitted light and 514 nm excited YFP images to define cellular structures for whole cell and lateral region measurements.

### 4.5 Heatmap generation based upon RNA sequence data

RNA sequence data were acquired from public repository (GEO accession number: http://www.ncbi.nlm.nih.gov/geo/query/acc.cgi?acc=GSE73336) (Engevik et al., 2016). Raw transcripts per million (TPM) from RNA sequence data were scaled to log_2_ scale. Significance tests are not considered for the organoid versus corpus tissue samples because the groups consist of single replicates.

### 4.6 Statistical analysis

All values are reported from experiments as the mean ± standard error of the mean (SEM) from 'n' organoid experiments. Statistical significance was determined using unpaired Student's T‐test or one‐way ANOVA with Dunnett's multiple comparison post hoc test. A *p* value of <.05 was considered significant.

## CONFLICT OF INTERESTS

The authors declare no competing or financial interests.

## AUTHOR CONTRIBUTIONS

K.A.E and M.H.M contributed to conceptualization: K.A.E also contributed to Methodology, Formal analysis, and Investigation and Y.O., R.A.K. contributed to Resources; Writing – the original draft: K.A.E. and M.H.M; Supervision: M.H.M.; Project administration: K.A.E. and M.H.M; and Funding acquisition: K.A.E., M.H.M.
